# Supplementary figures and images for: Characteristics and Expression Analysis of Invertase Gene Family in Common Wheat (Triticum aestivum L.)
Source: Genes (Basel). 2022 Dec 23;14(1):41. doi: 10.3390/genes14010041 (PMC9858860; doi:10.3390/genes14010041)

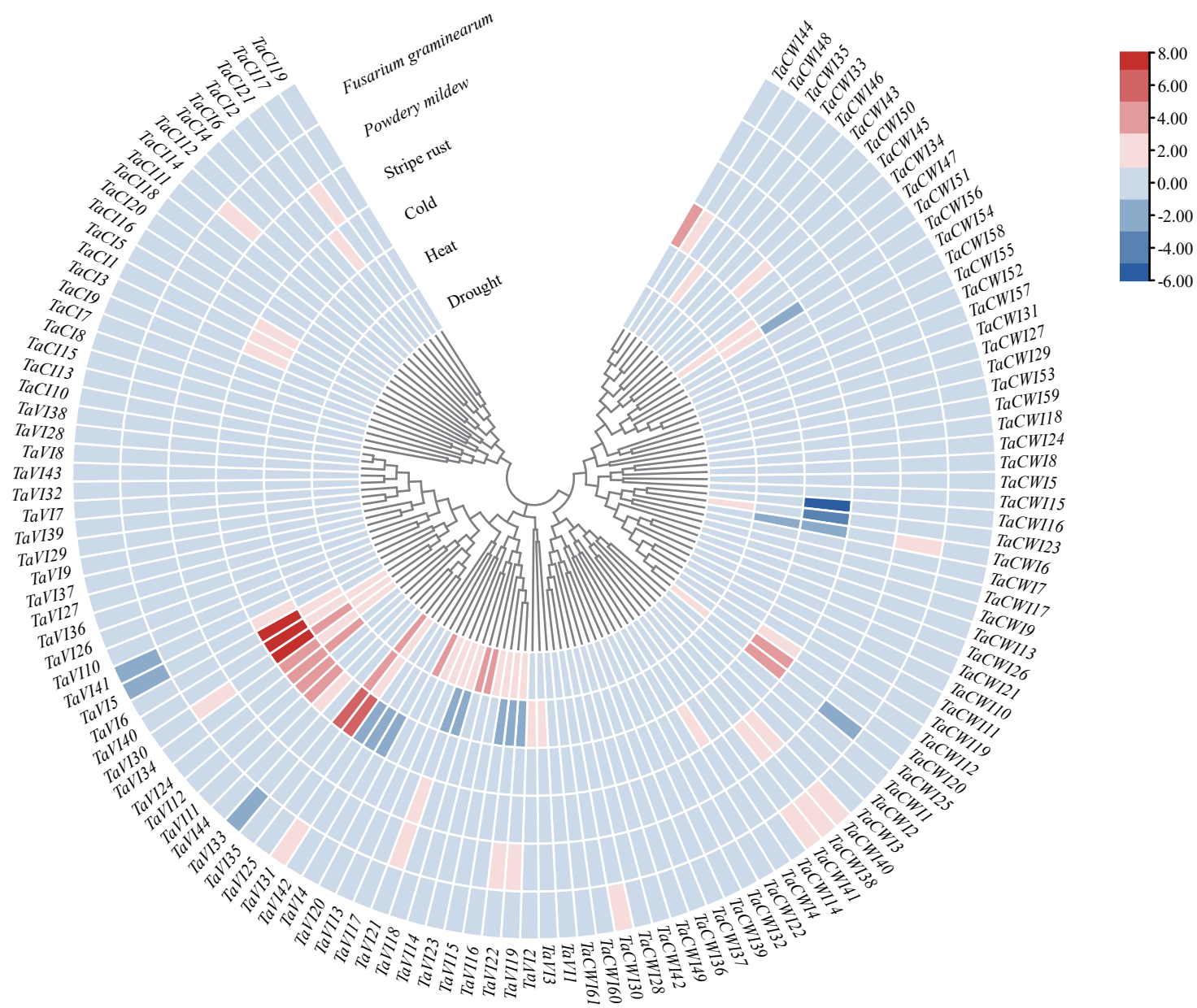

Supplement: Supplementary file 1 [file genes-14-00041-s001.zip › Figure S1.pdf]
